# Impacto del equipo multidisciplinario “ECMO Team” en el pronóstico de pacientes sometidos a membrana de oxigenación extracorpórea venoarterial por choque cardiogénico o paro cardiorrespiratorio refractario

**DOI:** 10.47487/apcyccv.v4i4.325

**Published:** 2023-12-27

**Authors:** Leonardo A. Seoane, Lucrecia Burgos, Rocío Baro Vila, Juan F. Furmento, Juan P. Costabel, Mariano Vrancic, Maximiliano Villagra, Olga D Ramírez-Hoyos, Daniel Navia, Mirta Diez

**Affiliations:** 1 Servicio de Cardiología Crítica, Departamento de Cardiología, ICBA Instituto Cardiovascular de Buenos Aires, Buenos Aires, Argentina. Servicio de Cardiología Crítica Departamento de Cardiología ICBA Instituto Cardiovascular de Buenos Aires Buenos Aires Argentina; 2 Sección de Insuficiencia Cardíaca, Departamento de Cardiología, ICBA Instituto Cardiovascular, Buenos Aires, Argentina. Sección de Insuficiencia Cardíaca Departamento de Cardiología ICBA Instituto Cardiovascular Buenos Aires Argentina; 3 Servicio de Cirugía cardiovascular, ICBA Instituto Cardiovascular de Buenos Aires, Buenos Aires, Argentina. Servicio de Cirugía cardiovascular ICBA Instituto Cardiovascular de Buenos Aires Buenos Aires Argentina; 4 Servicio de Ultrasonido, Departamento de Diagnóstico por Imagen. ICBA Instituto Cardiovascular de Buenos Aires Buenos AiresArgentina. Servicio de Ultrasonido Departamento de Diagnóstico por Imagen ICBA Instituto Cardiovascular de Buenos Aires Buenos Aires Argentina; 5 Sección de Perfusión, Servicio de Cirugía Cardiovascular, ICBA Instituto Cardiovascular de Buenos Aires, Buenos Aires, Argentina. Sección de Perfusión Servicio de Cirugía Cardiovascular ICBA Instituto Cardiovascular de Buenos Aires Buenos Aires Argentina

**Keywords:** Paro cardiaco, Grupo de Atención al Paciente, Oxigenación por Membrana Extracorpórea, Choque Cardiogénico, Heart Arrest, Patient Care Team, Extracorporeal Membrane Oxygenation, Shock, Cardiogenic

## Abstract

**Introducción.:**

La oxigenación por membrana extracorpórea venoarterial (ECMO VA) es una intervención de rescate utilizada en choque cardiogénico (CC) o paro cardiorrespiratorio (PCR) refractario. La creación de equipos multidisciplinarios *ECMO Teams* (ECMO T), ha permitido la estandarización de procesos, aunque se desconoce su impacto en sobrevida y pronóstico.

**Objetivo:**

El propósito es analizar si la creación del *ECMO Team* ha modificado el pronóstico de los pacientes sometidos a ECMO VA por CC o PCR refractario.

**Materiales y métodos.:**

Estudio observacional, unicéntrico, retrospectivo, que comparó los resultados del implante de ECMO VA por CC o PCR refractario en dos períodos consecutivos: entre 2014 y abril de 2019 (pre-*ECMO T*), y entre mayo de 2019 y diciembre de 2022 (pos-*ECMO T*). Como puntos finales, se evaluó la sobrevida intrahospitalaria y en ECMO, complicaciones, y volumen de ECMO anual.

**Resultados.:**

Se analizaron 83 pacientes (36 pre-*ECMO T*, y 47 pos-*ECMO T*), con edad de 53 +/-13 años. La causa más frecuente de asistencia fue: poscardiotomía pre-*ECMO T* (47,2%) y CC refractario pos-*ECMO T* (29,7%). En el 14,5% se realizó ECMO en PCR. La mediana de asistencia fue mayor pos-*ECMO T* (8 días, RIC 5-12,5 vs. 5 días, RIC 2-9 pre-*ECMO T*; p:0,04). La supervivencia al alta fue del 45,8% (38,9% pre-*ECMO T* vs. 51,1% pos-*ECMO T*;p:0,37) y en ECMO VA del 60,2% (55,6% pre-*ECMO T* y 63,8% pos-*ECMO T*; p:0,50). El volumen de ECMO VA fue significativamente mayor pos-*ECMO T* (13,2+/3,5 por año vs. 6,5+/-3,5 por año, p: 0,02). La tasa de complicaciones fue similar en ambos períodos.

**Conclusiones.:**

Luego de la implementación del *ECMO Team* no se observó una diferencia significativa en la sobrevida en pacientes asistidos con ECMO VA. Sin embargo, luego de su creación se evidenció un aumento significativo del volumen de pacientes asistidos por año. Pos-*ECMO T* se asistió mayor número de pacientes por choque cardiogénico, en PCR y con más días de asistencia.

## Abstract (en)

**Objectives.:**

Veno-arterial Extracorporeal membrane oxygenation (VA ECMO) is a salvage intervention in patients with cardiogenic shock (CS), and cardiac arrest (CA) refractory to standard therapies. The design of ECMO Teams has achieved the standardization of processes, although its impact on survival and prognosis is unknown.

**Objective:**

We aimed to analyze whether the creation of an ECMO Team has modified the prognosis of patients undergoing VA ECMO for refractory CS or CA.

**Materials and methods:**

. We conducted a single-center retrospective cohort study. Patients with refractory CS or CA who underwent VA ECMO were divided in two consecutive periods: from 2014 to April 2019 (pre-ECMO T) and from May 2019 to December 2022 (Post ECMO T). The main outcomes were survival on ECMO, in-hospital survival, complications, and annual ECMO volume.

**Results.:**

Eighty-three patients were included (36 pre-ECMO T and 47 post-ECMO T). The mean age was 53 +/-13 years. The most common reason for device indication was different: postcardiotomy shock (47.2%) pre-ECMO T and refractory cardiogenic shock (29.7%) post-ECMO T. The rate of extracorporeal cardiopulmonary resuscitation was 14.5%. The median duration of VA ECMO was longer after ECMO team implementation: 8 days (IQR 5-12.5) vs. five days (IQR 2-9, p=0.04). Global in-hospital survival was 45.8% (38.9% pre-ECMO T vs. 51.1% post-ECMO T; p=0.37), and the survival rate from VA ECMO was 60.2% (55.6% pre-ECMO T vs 63.8% post-ECMO T; p= 0.50). The volume of VA ECMO implantation was significantly higher in the post-ECMO team period (13.2 +/3.5 per year vs. 6.5 +/-3.5 per year, p: 0.02). The rate of complications was similar in both groups.

**Conclusions.:**

After the implementation of an ECMO team, there was no statistical difference in the survival rate of patients treated with VA ECMO. However, a significant increase in the number of patients supported per year was observed after the implementation of this multidisciplinary team. Post-ECMO T, the most common reason for device indication was cardiogenic shock, with longer run times and a higher rate of extracorporeal cardiopulmonary resuscitation.

## Introducción

La oxigenación por membrana extracorpórea venoarterial (ECMO VA) es un tipo de asistencia ventricular completa de flujo continuo, la cual proporciona soporte vital a pacientes con insuficiencia cardíaca, refractaria a las técnicas de soportes convencionales ^1^. Es considerada una intervención de rescate utilizada en pacientes con choque cardiogénico (CC) o paro cardiorrespiratorio (PCR) refractarios.

La mortalidad y las complicaciones asociadas a la oxigenación por membrana extracorpórea (ECMO) varía entre las distintas regiones, y los distintos centros, dependiendo de la infraestructura y experiencia de cada institución ^2^. Para ello, la Extracorporeal Life Support Organization (ELSO) ha desarrollado distintas guías clínicas que indican las recomendaciones para llevar a cabo este tipo de asistencia de manera estandarizada ^3^. Sin embargo, a pesar de las guías internacionales, de los avances en la tecnología asociada a ECMO, el mayor conocimiento y entrenamiento de los equipos tratantes, la mortalidad intrahospitalaria de los pacientes adultos asistidos en ECMO VA, según el registro ELSO, ha permanecido similar en las últimas décadas, variando entre el 50 y 60% ^4^.

La creación de equipos multidisciplinarios *ECMO Teams* (ECMO T), con la inclusión de médicos de diferentes especialidades críticas, enfermeros, perfusionistas y kinesiólogos, ha permitido la estandarización de procesos, y selección adecuada de los pacientes. Considerando la complejidad de esta población, las guías de la ELSO y múltiples consensos recomiendan el abordaje interdisciplinario a través de equipos de expertos ^5^. En paralelo, existe cierta evidencia de que estos grupos multidisciplinarios podrían asociarse a mejores resultados de los pacientes en ECMO ^6^^-^^8^. Sin embargo, la mayoría de estos estudios se desarrollaron en pacientes con insuficiencia respiratoria refractaria asistidos con ECMO venovenoso, no se llevaron a cabo en Latinoamérica y no demostraron un beneficio claro en términos de mortalidad ^7^^-^^9^. Por lo tanto, se desconoce si la creación de estos equipos multidisciplinarios tiene un impacto real en la sobrevida y el pronóstico de los pacientes en ECMO VA.

El propósito del presente trabajo es analizar si la creación del equipo multidisciplinario *ECMO Team* modifica el pronóstico y la evolución de los pacientes sometidos a ECMO VA o el volumen de asistencia por CC o PCR refractario en un centro monovalente cardiovascular de Argentina.

## Materiales y métodos

### Diseño y población de estudio

Estudio retrospectivo, observacional, transversal y unicéntrico. Se analizó la base de datos institucional de asistencia ventricular, la cual es completada prospectivamente desde 2014. Se evaluaron las características demográficas, clínicas, información del tipo de asistencia ventricular, complicaciones y eventos clínicos de relevancia. Para el análisis se los dividió en dos períodos definidos por la creación del equipo multidisciplinario *ECMO Team*, que ocurrió en mayo de 2019.

Fueron elegibles para su inclusión todos los pacientes consecutivos con edad mayor o igual a 18 años; con implante de ECMO VA, con canulación central y/o periférica, a los cuales se les indicó por CC o PCR refractario. Se definió al CC refractario como todo choque de causa cardíaca con requerimiento de dos o más drogas inotrópicas a dosis intermedias/altas (ej: noradrenalina a 0,5 mcg/kg/min). Por su parte, el PCR refractario se definió como PCR presenciado, de probable causa cardíaca (principalmente con taquicardia ventricular o fibrilación ventricular como ritmo de inicio), que se extiende más de 10 min, aun con una adecuada reanimación cardiopulmonar desde el inicio de este.

Se excluyeron aquellos pacientes a los cuales se les implantó ECMO venovenoso (ECMO VV) por insuficiencia respiratoria refractaria, u otro tipo de asistencia ventricular completa distinta al ECMO VA, como por ejemplo Centrimag.

### Procedimientos

El *ECMO Team*, si bien forma parte de la clínica de trasplante y asistencia ventricular, se encuentra conformado por especialistas de diversos servicios: cirujanos cardiovasculares, cardiólogos críticos con especialidad en terapia intensiva, cardiólogos especializados en insuficiencia cardíaca, perfusionistas, enfermeros, kinesiólogos, cardiólogos especialistas en ultrasonido, anestesiólogos, cardiólogos intervencionistas, nutricionistas, hematólogos e infectólogos. El *ECMO team* es parte del *Shock team*, simplemente focalizado en el ECMO VA como soporte circulatorio. Los actores principales del equipo (cirujanos cardiovasculares, perfusionistas, cardiólogos y enfermeros) deben estar activos las 24 h del día durante los 365 días del año para realizar una eventual canulación de urgencia, o para resolver complicaciones del ECMO lo más rápido posible. 

Los objetivos principales del diseño de un *ECMO Team* fueron estandarizar los procesos, determinar los criterios unificados de inclusión y exclusión para el implante del ECMO VA, y lograr una asistencia ventricular en el tiempo adecuado en los casos de choque cardiogénico refractario. Posterior al implante se encarga de realizar un seguimiento y monitoreo diario del paciente y determinar el tiempo y el modo de destete de la asistencia Se focalizó inicialmente en la realización de *checklists* para el armado, purgado e implante del ECMO, y en la norma de activación del equipo. Se continuó luego con protocolos para el implante percutáneo y quirúrgico, manejo de anticoagulación, utilización del ultrasonido (para implante, seguimiento y destete), profilaxis de infecciones, nutrición y cuidado de enfermería. Luego de la maduración del equipo se focalizó en la realización de ECMO VA en paro cardiorrespiratorio intrahospitalario, y en ECMO de traslado, para aumentar el volumen de asistencia. 

La realización del *ECMO Team*, además de la organización y estandarización de procesos, incluyó un aprendizaje teórico como un entrenamiento de habilidades psicofísicas. Se realizaron cursos de capacitación en ECMO, con simulación de escenarios de emergencia. Posteriormente se diseñaron desde la institución cursos de simulación de alta fidelidad para personal externo e interno, con una frecuencia mínima de dos veces por año.

Como el *ECMO Team* se diseñó en mayo de 2019, se dividió a la población en estudio en dos períodos comparables: entre enero de 2014 y abril de 2019 (pre-ECMO T) y un segundo grupo de asistidos entre mayo de 2019 y diciembre de 2022 (pos-ECMO T).

### Variables de estudio

Dentro de las variables clínicas y principales complicaciones a analizar se incluyeron las siguientes:

- Sobrevida en ECMO VA. Evalúa la supervivencia en ECMO, y hasta las 24 h del destete de la asistencia ventricular. En este caso, los motivos de desvinculación del ECMO son por recuperación de la función cardíaca o porque se realizó un trasplante cardíaco.
- Sobrevida al alta. Evalúa la supervivencia al alta hospitalaria, ya sea por alta sanatorial o derivación a otro centro sanitario (ej. tercer nivel de rehabilitación).
- Volumen anual de ECMO VA. Media de implantes de ECMO VA por año en cada período.
- Complicaciones mecánicas. Son aquellas propias de la asistencia, que requieren intervención como el cambio del equipo o los componentes del circuito del ECMO. Incluyen el fallo de membrana, fallo del cono, ruptura de tubuladuras, cambio del circuito ya sea por aire o trombos en el mismo, y disfunción del regulador de temperatura.
- Complicaciones hemorrágicas. Son los sangrados que requieren transfusión >20 mL/kg/día o >3 unidades de glóbulos rojos por día.
- Complicaciones neurológicas. Muerte cerebral (pérdida irreversible de la conciencia, sumado a la pérdida irreversible de las funciones neurovegetativas, incluida la capacidad de respirar) y accidente cerebrovascular (foco neurológico agudo y cambios isquémicos o hemorrágicos nuevos en la tomografía de cerebro).
- Complicaciones infecciosas. Infección constatada previo al implante o en ECMO, con o sin rescate microbiológico, con requerimiento de antimicrobianos.
- Complicaciones tromboembólicas. Presencia de trombosis o embolias constatadas en el paciente (ya sea clínicas o imagenológicas) o el ECMO.
- Complicaciones renales. La insuficiencia renal se define como el cambio de creatinina luego del implante del ECMO (alcanzando una creatinina mayor o igual a 1,5 mg/dL) o el requerimiento de diálisis.

### Análisis estadístico

La distribución paramétrica de las variables continuas cuantitativas se evaluó mediante la prueba de Kolmogorov-Smirnov. Las variables cualitativas se expresaron en proporciones, mientras que las cuantitativas continuas en medias con sus respectivos desvíos estándar (DE) (en caso de distribución paramétrica) o medianas y rango intercuartilo para las no paramétricas.

Se utilizó la prueba de T de Student para el análisis de variables cuantitativas de distribución paramétrica y la prueba de Mann-Whitney U en caso de no paramétricas. La asociación entre variables cualitativas se definió a partir de las pruebas de chi cuadrado y la prueba de Fisher. Se consideró un error alfa a dos colas del 5% como valor estadísticamente significativo (p<0,05).

Los análisis estadísticos fueron realizados utilizando el *Software* IBM SPSS Statistics (versión 22, SPSS, IBM Corporation, Armonk, New York).

### Consideraciones éticas

Este estudio cumple todos los requerimientos contenidos en el código ético de la OMS (Declaración de Helsinki), fue aprobado por el Comité de Investigación clínica del Instituto y el comité de ética. Todos los pacientes firmaron el *habeas data*.

## Resultados

Se analizaron 83 pacientes consecutivos a los que se les implantó ECMO VA por CC o PCR refractarios. De ellos, 36 fueron previo a la creación del *ECMO Team* (pre-*ECMO T*) y 47 posterior al mismo (pos-*ECMO T*). La media de edad de la población global fue de 53 años (DE:13,0), la mayoría de sexo masculino (57,8%). Respecto a los factores de riesgo cardiovascular, el 47,0% presentaba dislipemia, el 34,9% hipertensión arterial y el 20,5% diabetes. La media de fracción de eyección poblacional fue del 28% (DE:12,8). El diagnóstico principal fue miocardiopatía isquémica necrótica (37,3%), seguido de enfermedad valvular significativa (21,7%). No se observaron diferencias significativas en las características poblacionales entre ambos períodos (Tabla 1).

**Tabla 1:** Características basales de la población incluida

Variables	Global (n: 83)	Pre-ECMO T (n: 36)	Pos-ECMO T (n: 47)	p
Edad en años, media (DE)	53 (13)	55 (13)	51 (13)	0,19
Sexo masculino, n (%)	48 (57,8)	24 (66,7)	24 (51,1)	0,18
IMC en kg/m^2^, media (DE)	26 (5)	26 (4)	26 (5)	0,96
HTA, n (%)	29 (34,9)	11 (30,6)	18 (38,3)	0,49
Diabetes, n (%)	17 (20,5)	8 (22,2)	9 (19,1)	0,98
Dislipemia, n (%)	39 (47,0)	20 (55,6)	19 (40,4)	0,27
Tabaquismo actual, n (%)	11 (13,3)	3 (8,3)	8 (17,0)	0,33
Enfermedad coronaria previa, n (%)	31 (37,3)	12 (33,3)	19 (40,4)	0,63
Enfermedad valvular moderada-severa, n (%)	18 (21,7)	8 (22,2)	10 (21,3)	0,98
Cirugía cardiaca previa, n (%)	10 (12,1)	6 (16,7)	4 (8,5)	0,33
ACV/AIT, n (%)	2 (2,4)	2 (5,6)	0 (0,0%)	0,19
EPOC, n (%)	2 (2,4)	1 (2,8)	1 (2,1)	0,99
ERC, n (%)	17 (20,5)	8 (22,2)	9 (19,1)	0,98
Anemia, n (%)	12 (14,5)	5 (13,9)	7 (14,9)	0,96
Fibrilación auricular, n (%)	15 (18,1)	9 (25,0)	6 (12,8)	0,25
FEVI previa <40%, n (%)	40 (48,2)	20 (55,5)	20 (42,6)	0,64

DE: desviación estándar. HTA: hipertensión arterial. IMC: índice de masa corporal. ACV: accidente cerebrovascular. AIT: accidente isquémico transitorio. EPOC: enfermedad pulmonar obstructiva crónica. ERC: enfermedad renal crónica. FEVI: fracción de eyección del ventrículo izquierdo.

Las indicaciones principales para el implante fueron diferentes para cada grupo: poscardiotomía pre-*ECMO T* (47,2%), y CC refractario (clínico, principalmente por infarto agudo de miocardio) pos-*ECMO T* (29,7%), p:0,04. El 14,5% se realizó ECMO VA en PCR (ECPR), siendo más frecuente pos-*ECMO T* (21,3% vs. 5,6%, p:0,04). 

La mayoría de los ECMO VA se indicaron en Intermacs 1 (78,3%), seguido por aquellos en Intermacs 2 (14,5%). Según la clasificación de SCAI, se implantó en estadio D en el 69,9% de los casos (n:58); SCAI E en el 18,1% (n:15), y estadio C en el 12% (n:10). Según los diferentes períodos, pre-*ECMO T* se observó la indicación de asistencia en SCAI D en el 66,7% (n:24); en estadio E en 27,8% (n:10), y en estadio C en el 5,6% de los casos (n:2). Posterior al diseño del equipo multidisciplinario el implante fue en SCAI D en 78,7% (n:37); estadio E en el 6,4% (n:3), y en estadio C en el 14,9% (n:7); observándose en este último período mayor porcentaje en estadio C, y menor en estadio terminal SCAI E. La canulación fue periférica en el 84,3%, (predominantemente fémoro-femoral), siendo central solo en el 15,7% de los casos, principalmente en pacientes poscardiotomía. 

Se requirió descompresión ventricular con técnicas de venteo (septostomía, cánula aferente extra por abordaje transeptal con drenaje de cavidades izquierdas, o drenaje de vena pulmonar) en el 19,8% de los pacientes, siendo mayor pos-*ECMO T* (31,9% vs. 2,8% pre-*ECMO T*; p<0,01). El 85,5% presentaba concomitantemente un balón de contrapulsación intraaórtico para favorecer la apertura valvular aórtica. La utilización global de catéter de Swan Ganz fue del 72,2% preimplante de ECMO VA (72,2% pre-*ECMO T* y 72,3% pos-*ECMO T*) y 48,2% luego del explante de la asistencia (41,7% pre-*ECMO T*, y 53,2% pos-*ECMO T*). La mediana de asistencia fue de 6,0 días (RIC: 3-10), siendo significativamente mayor pos-*ECMO T* (8 días, RIC 5-12,5 vs. 5 días, RIC: 2-9 en pre-*ECMO T*; p:0,04); (Tabla 2). El ECMO VA de mayor duración fue de 26 días.

**Tabla 2:** Características clínicas relacionadas con la asistencia ventricular

Variables	Global (n: 83)	Pre-ECMO T (n: 36)	Pos-ECMO T (n: 47)	p
Enfermedad de base, n (%)				0,63
Dilatada idiopática	6 (7,2)	3 (8,3)	3 (6,4)	
Isquémico necrótica	31 (37,3)	12 (33,3)	19 (40,4)	
Valvular	18 (21,7)	8 (22,2)	10 (21,3)	
MCH	4 (4,8)	3 (8,3)	1 (2,1)	
Miocarditis	5 (6,0)	1 (2,8)	4 (8,5)	
MNC	4 (4,8)	3 (8,3)	1 (2,1)	
Otras	11 (13,4)	4 (11,1)	7 (14,9)	
Chagas	4 (4,8)	2 (5,6)	2 (4,3)	
Indicaciones del implante, n (%)				
Poscardiotomía	28 (33,7)	17 (47,2)	11 (23,4)	0,04
Choque cardiogénico	22 (26,5)	8 (22,2)	14 (29,7)	
Falla primaria del injerto	17 (20,5)	8 (22,2)	9 (19,2)	
PCR	12 (14,5)	2 (5,6)	10 (21,3)	
Tormenta eléctrica	4 (4,8)	1 (2,8)	3 (6,4)	
ECMO en PCR, n (%)	12 (14,5)	2 (5,6)	10 (21,3)	0,04
INTERMACS, n (%)				
1	65 (78,3)	31 (86,1)	34 (72,3)	0,65
2	12 (14,5)	4 (11,1)	8 (17,0)	
3	6 (7,2)	1 (2,8)	5 (10,7)	
Estrategia de implante, n (%)				
Puente a trasplante	22 (26,5)	10 (27,7)	12 (25,5)	0,99
Puente a recuperación	56 (67,5)	24 (66,7)	32 (68,1)	
Puente a decisión	5 (6,0)	2 (5,6)	3 (6,4)	
Puente a puente	0 (0,0)	0 (0,0)	0 (0,0)	
BCIAo en ECMO, n (%)	71 (85,5)	31 (86,1)	40 (85,1)	0,82
Levitronix® CentriMag previa, n (%)	2 (2,4)	1 (2,8)	1 (2,1)	0,99
Canulación periférica, n (%)	70 (84,3)	31 (86,1)	39 (83,0)	0,77
Venteo ventricular, n (%)	16 (19,8)	1 (2,8)	15 (31,9)	<0,01
Duración en días, mediana (RIC)	6 (3-10)	5 (2-9)	8 (5-12,5)	0,04

BCIAo: balón de contrapulsación intraaórtico. MCH: miocardiopatía hipertrófica. MNC: miocardio no compacto. PCR: paro cardiorrespiratorio. ECMO: membrana de oxigenación extracorpórea. RIC: rango intercuartilo.

La tasa de supervivencia global al alta fue del 45,8%, siendo de 38,9% previo a la creación del *ECMO Team*, y de 51,1% pos-*ECMO Team* (p:0,37). Por su parte, la sobrevida global en ECMO VA fue del 60,2%, siendo mayor en el período post ECMO Team, pero sin alcanzar la significancia estadística (55,6% pre-*ECMO T* vs. 63,8% pos-*ECMO T*; p:0,50). En contraposición, el volumen de ECMO VA fue significativamente mayor en el período pos-*ECMO T* (13,2 casos+/3,5 por año vs. 6,5+/-3,5 por año, p:0,02); (Tabla 3).

**Tabla 3:** Sobrevida, volumen de asistencia, y complicaciones asociadas al ECMO VA

Variables	Global (n: 83)	Pre-ECMO Team (n: 36)	Pos-ECMO Team (n: 47)	p
Sobrevida IH, n (%)	38 (45,8)	14 (38,9)	24 (51,1)	0,37
	50 (60,2):			
Sobrevida en ECMO, n (%)	Rec: 38 (45,7)	20 (55,6)	30 (63,8)	0,50
	Tx: 12 (14,5)			
Volumen de ECMO/año, media (DE)	9,2 (3,2)	6,5 (3,5)	13,2 (3,5)	0,02
IRA, n (%)	42 (50,6)	22 (61,1)	20 (42,6)	0,10
Sangrado, n (%)	49 (59,0)	23 (63,9)	26 (55,3)	0,46
Infección, n (%)	40 (48,2)	19 (52,8)	21 (44,6)	0,82
Trombosis, n (%)	37 (44,6)	16 (44,4)	21 (44,7)	0,90
Complicación mecánica, n (%)	0 (0,0)	0 (0,0)	0 (0,0)	0,99
Muerte cerebral, n (%)	1 (1,2)	1 (2,8)	0 (0,0)	0,46
ACV, n (%)	10 (12,0)	6 (16,7)	4 (8,5)	0,50
Requerimiento de diálisis, n (%)	23 (27,7)	10 (27,8)	13 (27,7)	0,99
Taponamiento, n (%)	17 (20,5)	6 (16,7)	11 (23,4)	0,42
AVM prolongada con traqueostomía, n (%)	32 (38,6)	9 (25,0)	23 (48,9)	0,01
Isquemia arterial periférica, n (%)	17 (20,5)	9 (25,0)	8 (17,0)	0,59

ACV: accidente cerebrovascular. AVM: asistencia ventilatoria mecánica. ECMO: membrana por oxigenación extracorpórea. IH: intrahospitalaria. IRA: insuficiencia renal aguda. Rec: recuperación. Tx: trasplante cardíaco.

Respecto al volumen de trasplante cardíaco, de manera global en nuestra institución pre-*ECMO Team* se realizaron 48 injertos durante dicho período (media de 9,1 casos por año) y 52 posterior al diseño del equipo (14,4 Casos por año). De los 36 pacientes en ECMO VA pre-*ECMO T*, se trasplantaron 15 (siendo el 41,7% de pacientes en ECMO VA, y el 31,2% del total de trasplantes cardíacos de ese período). En cambio, pos-ECMO T se trasplantaron 14 pacientes asistidos previamente en ECMO VA, correspondiendo al 29,8% de pacientes en ECMO VA, y al 26,9% del total de trasplantes de dicho período. En cuanto al implante de dispositivos de largo plazo, no se realizó ninguno ya que no están disponibles en Argentina. 

Las principales complicaciones durante la asistencia en ECMO VA fueron el sangrado (59,0%), la insuficiencia renal (50,6%), las infecciones (48,2%) y los eventos tromboembólicos (44,6%), siendo similar en ambos períodos. La mayoría de los sangrados fueron médicos, principalmente del sitio de implante de ECMO. La principal complicación vascular fue la isquemia arterial periférica, que fue del 20,5% (n:17), siendo similar en ambos períodos (25,0% pre-*ECMO Team* vs. 17,0% pos-*ECMO Team*, p:0,59); **(**Tabla 3**)**. Se observó sangrado del sitio de canulación periférica en el 46,9% de los casos (50,0% pre-*ECMO T* vs. 44,6% pos-*ECMO T*). Se realizó fasciotomía en 6,0% de los casos (n:5), siendo similar en ambos períodos (5,5% pre-*ECMO T* vs. 6,4% pos-*ECMO T*). Solo se observaron dos amputaciones de miembro inferior infrapatelar por isquemia arterial correspondiendo al 2,4% del total (1 pre y 1 pos-*ECMO Team*). No se objetivaron complicaciones mecánicas asociadas a la ECMO en ninguno de los dos períodos (fallo de membrana o cono, ruptura de tubuladuras o disfunción de regulador de temperatura).

Con relación al resto de las complicaciones, la única que fue significativamente distinta en alguno de los dos períodos fue la asistencia mecánica ventilatoria prolongada con requerimiento de traqueostomía, que fue mayor poscreación del *ECMO Team*, (48,9% vs. 25,0% pre-*ECMO T*, p:0,01); (Tabla 3).

Respecto a los pacientes con infarto agudo de miocardio, ninguno presentó complicaciones mecánicas asociadas al infarto, y se requirió descompresión de cavidades izquierdas con septostomía en el 35% de los casos. Si bien los pacientes presentaban doble antiagregación y anticoagulación, la tasa de sangrado fue del 37,5%, siendo menor a la serie global.

## Discusión

**Figura 1:**
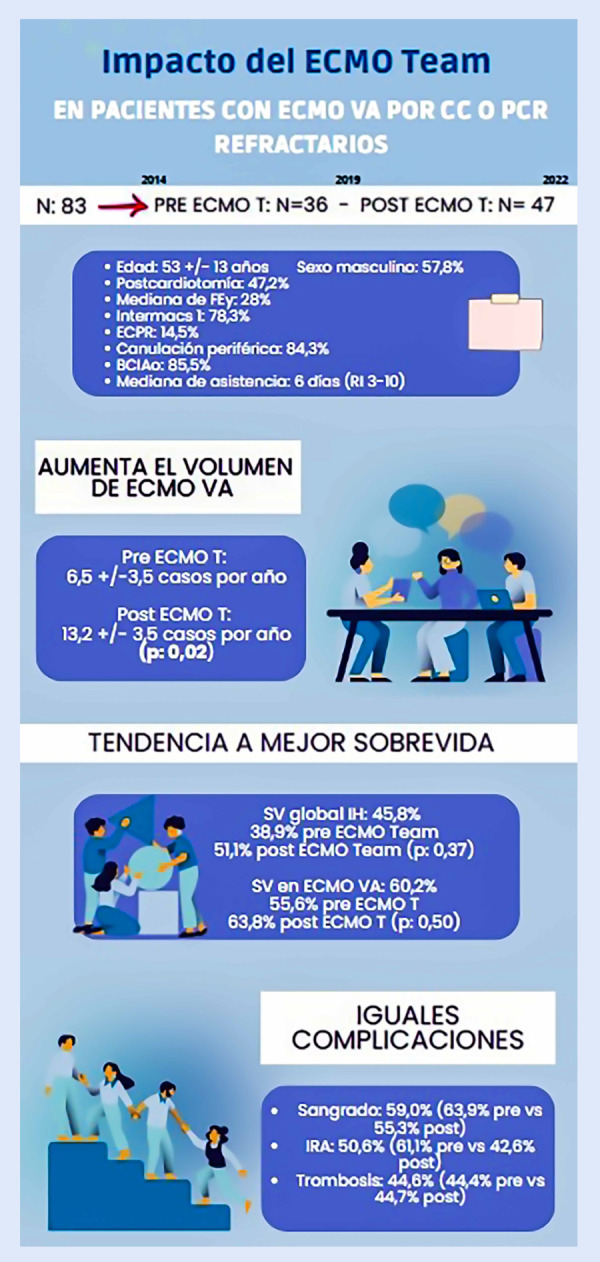
Infografía resumen del estudio

El ECMO VA es un tipo de asistencia ventricular de corto plazo cada vez más desarrollada a nivel mundial, utilizada para el manejo de CC y el PCR refractario, incluso en países subdesarrolladas, siendo en este caso prácticamente la única disponible. El desarrollo de equipos multidisciplinario para la decisión del implante y el manejo de estos pacientes es fundamental, y parecería mejorar el pronóstico y los eventos clínicos. Sin embargo, en nuestro estudio luego de la creación del *ECMO Team* no se observó un aumento significativo del destete de ECMO VA, ni una mejora en la sobrevida. De todas maneras, se objetivó un aumento significativo del volumen de pacientes asistidos por año en período pos-*ECMO T* **(**Figura 1**).**

BCIAo: balón de contrapulsación intraaórtico. ECMO T: ECMO Team. ECMO VA: oxigenación por membrana extracorpórea venoarterial. ECPR: ECMO en paro cardiorrespiratorio. FEy: fracción de eyección. IRA: insuficiencia renal aguda. PCR: paro cardiorrespiratorio. CC: choque cardiogénico. SV: sobrevida.

Existen algunos trabajos que evaluaron el impacto de un equipo multidisciplinario en pacientes en ECMO, pero presentando cierta heterogeneidad tanto en el diseño, la población seleccionada, como en los resultados. El estudio de Komindr *et al*. incluyó 69 pacientes, siendo la gran mayoría ECMO VA (94%) y al igual que en nuestra experiencia no hubo una variación en la mortalidad en ambos períodos, ni en la estancia hospitalaria. Al igual que en nuestro trabajo, se observó mayor número de asistencias (22,7 pre-*ECMO T* vs. 36,3 pos-*ECMO T*) y mayor recopilación de datos ^9^. Probablemente, la diferencia en mortalidad no fue significativa ya que el centro japonés presentaba alto volumen, con gran experiencia desde el inicio del estudio (inclusive en el primer período), presentando más de 20 ECMO por año y reflejado también en la alta sobrevida global (67%, comparada con el 46% descripta en el registro ELSO) ^4^.

Por el contrario, Dalia *et al.* demostraron con mayor número de pacientes (n: 279) que posterior al diseño del *ECMO Team*, se observó un aumento significativo de la sobrevida intrahospitalaria (37,7% vs. 52,3%, p: 0,02) ^6^. De todas maneras, en el primer período se realizaron significativamente más ECMO VA, en comparación con el segundo período (76% vs. 51%) y menos venovenoso (19,2% vs. 30,8%, respectivamente). Se conoce según el registro ELSO que la mortalidad del paciente adulto en ECMO VA es mayor al del ECMO VV (54% vs. 42%, respectivamente) por lo que se estaría comparando en lugar de dos períodos diferentes, dos asistencias distintas (cardíaca vs. respiratoria), con distinto grado de mortalidad ^4^. En el mismo sentido, cuando se compara solo la subpoblación que recibió ECMO VA, no se observó una diferencia en términos de sobrevida. Cotza *et al*. diseñaron en el Policlínico San Donato de Italia un estudio similar, incluyendo 100 pacientes en ECMO, tanto VA (67,4%) como VV, adultos (52%) y pediátricos ^10^. Se evidenció en este caso que la mortalidad fue menor en el período pos-*ECMO T* (44% vs. 62,5%, respectivamente). De todas maneras, no se realizaron pruebas estadísticas para determinar la significancia en dicho estudio, por lo que se desconoce si es significativa esa diferencia. 

Existen otros estudios que han demostrado el beneficio en términos de sobrevida del *ECMO Team*, pero todos realizados en otras regiones y con pacientes asistidos en ECMO VV por insuficiencia respiratoria refractaria. Entre ellos Goh *et al*. quienes demostraron que posterior al diseño de un equipo multidisciplinario guiado por un intensivista se redujo la mortalidad del 44,4 al 14,8% en pacientes asistidos en ECMO VV ^7^. Fue un estudio retrospectivo que incluyó 108 pacientes en un centro de Singapur y la diferencia fue significativa aun luego del ajuste a diferentes variables confundidoras. Es interesante recordar que estos pacientes con insuficiencia respiratoria refractaria presentan menor mortalidad que el choque cardiogénico refractario en ECMO VA. De todas maneras, luego de la implementación del *ECMO Team* se alcanzaron valores extremadamente bajos de mortalidad intrahospitalaria, en relación a los descriptos por la ELSO en adultos en ECMO VV (14,8% vs. 42%, respectivamente)^4^. En la misma línea, Na *et al*. demostraron en su cohorte de 116 pacientes asistidos en ECMO VV en Corea del Sur, que posterior al diseño del *ECMO Team* se observó una reducción significativa de la mortalidad intrahospitalaria del 75,7 al 52,2% ^8^. De todas maneras, estos valores son excesivos en comparación al registro ELSO o al trabajo enunciado previamente de Goh *et al*., por lo que es más sencillo mostrar una mejora partiendo de valores de mortalidad tan elevados.

Probablemente esta diferencia en términos de reducción de mortalidad intrahospitalaria, luego del diseño del *ECMO Team* entre los pacientes asistidos por insuficiencia respiratoria o choque cardiogénico, se deba a que los trabajos diseñados exclusivamente con ECMO VV incluyeron mayor número de pacientes, o probablemente a que sea más fácil estandarizar procesos en pacientes menos complejos como puede ser un *distress* respiratorio, obteniendo mejoras rápidamente.

Es importante destacar que el diseño del *ECMO Team* en nuestro estudio ha aumentado el número de pacientes asistidos en ECMO VA por año. Esto permite que pacientes en CC o PCR tengan la posibilidad de sobrevivir, ya sea como puente a recuperación o trasplante cardíaco, que sería imposible sin este tipo de asistencia ^9^. Adicionalmente, existe evidencia de que el mayor volumen de pacientes asistidos en ECMO en un centro se asocia a menor mortalidad intrahospitalaria. Barbaro *et al*. analizaron a partir del Registro ELSO más de 55 000 pacientes en ECMO VV y VA (39%) y demostraron que en los tres grupos etarios el alto volumen de asistencias en ECMO por año, redujo significativamente la mortalidad intrahospitalaria en dichas instituciones ^11^. Los pacientes adultos en ECMO atendidos en hospitales con más de 30 casos anuales se asociaron con menor mortalidad intrahospitalaria, en comparación con los atendidos en los centros con menos de 6 casos anuales (OR 0,61,IC 95% 0,46-0,80). Tchantchaleishvili *et al*. en su institución en Birmingham demostraron también, al pasar de ser un centro con bajo volumen de ECMO a un alto volumen, presentar mejoría en la sobrevida intrahospitalaria ^12^.

En contraposición, Komindr *et al.*, no pudieron evidenciar esta mejoría de sobrevida (incluso aumentando el volumen de ECMO) luego de la creación del *ECMO Team*, pero probablemente porque ya partían de un volumen elevado de pacientes en ECMO por año, por lo que estarían comparando dos períodos similares. En nuestra experiencia, al partir de una media de menos de siete casos por año (bajo volumen) y alcanzar luego del diseño del equipo multidisciplinario una media de entre 10 y 20 corridas de ECMO por año (volumen moderado), probablemente el beneficio sea mayor. En nuestro estudio observamos una mejoría en la sobrevida intrahospitalaria (del 38,9 al 51,1%),y de sobrevida en ECMO (de 55,6 al 63,8%), pero sin alcanzar la significancia estadística. Probablemente el bajo número de pacientes analizado pudo influir en el resultado. Sumado a ello, pos-*ECMO T* se incluyó mayor porcentaje de pacientes con ECPR, con menor sobrevida conocida (30% en ECPR vs. 46% en ECMO VA cardíaco) y el motivo de asistencia fue predominantemente por CC refractario posinfarto (menos tasa de poscardiotomía) probablemente con menor tasa de reversibilidad de la causa del choque, reflejado en asistencias significativamente más largas. De todas maneras, se alcanzaron valores de sobrevida en ECMO e intrahospitalarios comparables al registro ELSO (51,1% de sobrevida intrahospitalaria vs. 46% en ELSO y 63,8% de sobrevida en ECMO vs. 60% en el registro internacional) ^4^.

De manera más amplia, los *Shock Teams* como equipos de abordaje multidisciplinario del choque cardiogénico han logrado reducir la mortalidad, favorecer la revascularización temprana en el infarto agudo de miocardio e indicar la asistencia temprana ^13^^-^^15^. Probablemente esto se deba a la adecuada selección de pacientes, el tipo de asistencia de corto plazo y la optimización de los tiempos a través de un equipo de expertos en medicina crítica ^16^. En este escenario clínico, Taleb *et al*. compararon 123 pacientes consecutivos con CC refractario a cargo de un *Shock Team* con 121 pacientes manejados con un algoritmo habitual, y evidenciaron en el primer grupo una reducción del riesgo absoluto del 13,1% en mortalidad intrahospitalaria, con reducción de mortalidad de todas las causas a 30 días con un HR de 0,61 (IC 95% 0,41-0,93) ^17^.

Respecto a los otros eventos clínicos analizados, en nuestra experiencia no hubo diferencias en las complicaciones en ECMO en ambos períodos, a excepción de la ventilación mecánica con requerimiento de traqueostomía, que fue significativamente mayor pos-*ECMO T*. Probablemente esto se deba a que en el segundo período se asistieron pacientes más complejos, con mayor porcentaje de ECPR y con asistencias significativamente más largas. Otra de las causas puede ser que con la protocolización y estandarización de procesos se realizaron más traqueostomías de manera precoz. En contraposición a nuestro estudio, Na *et al*. han demostrado reducir en pacientes asistidos en ECMO VV la incidencia de problemas asociados a la cánula (32,9% vs. 15,2%, p:0,034) y los eventos cardiovasculares (88,6% vs. 65,2% p:0,002) luego de la implementación del ECMO Team ^8^.

El desarrollo de un equipo multidisciplinario para el manejo de pacientes en ECMO es un desafío, y aun más en países subdesarrollados. Se necesita en algunos casos mínimamente un año y medio para diseñar e implementar un programa de ECMO ^18^. Posteriormente, es clave el entrenamiento continuo, más aun en centros con bajo volumen de asistencia por año, donde los resultados tienden a ser desfavorables. En contraposición, Nagaoka *et al*. han demostrado presentar resultados adecuados con la creación de un equipo multidisciplinario para el manejo de pacientes en ECMO VV por COVID-19 en un centro de bajo volumen en Japón ^19^. Luego de asistir tan solo cinco pacientes, el 80% sobrevivió con el abordaje del *ECMO Team*. De todas maneras, es un número muy pequeño de pacientes como para llegar a una conclusión, además de ser asistidos por insuficiencia respiratoria. Por su parte, Assy *et al*. han podido desarrollar un programa de ECMO en un país subdesarrollado como es Líbano, y con resultados óptimos. Desde 2015 hasta 2018 han asistido a 12 pacientes en ECMO, siendo la mayoría venoarterial, con sobrevida en ECMO del 75% y al alta de 41% ^20^. En comparación, en nuestro estudio, hemos incluido 83 asistencias en ECMO VA por CC y PCR refractario, por lo que es un número considerable para un país de bajos ingresos de Latinoamérica, considerando además que la mediana de asistencias por centro por año en dicha región es de 5 a 6 pacientes según el Registro ELSO ^4^.

Como significancia clínica, es el primer estudio llevado a cabo en un país subdesarrollado de Sudamérica en comparar la experiencia de ECMO VA pre y posdiseño de un equipo multidisciplinario. Se ha demostrado que, aun en un país donde existen recursos y acceso limitado a los dispositivos de asistencia ventricular compleja, el diseño e implementación de un *ECMO Team* es factible y permite obtener resultados óptimos comparables a la experiencia internacional, con mejora no significativa de la sobrevida y con aumento del volumen de asistencia anual. Desde un modelo conceptual, el mayor volumen de ECMO permite estructurar y estandarizar los procesos de cuidado (conocimiento y entrenamiento del personal, desarrollo de protocolos, selección adecuada del paciente) lo que llevaría a mejora en la calidad del cuidado del paciente en ECMO, con una consecuente reducción de la mortalidad, por lo que el centro comenzaría a ser referente tanto a nivel local y regional, aumentando así el volumen de corridas de ECMO anuales, completando así el círculo virtuoso ^11^. La presencia de un *ECMO Team* activo institucional, con reuniones periódicas (inicialmente semanales), revisión y creación de nuevos protocolos, discusión de casos y entrenamiento continuo del personal, permitirá seguramente mantener en el tiempo la calidad de atención y la optimización de resultados de los pacientes en ECMO VA.

Este estudio tiene ciertas limitaciones que es preciso mencionar. En primer lugar, es un estudio observacional retrospectivo, con los sesgos que ello acarrea. En segundo lugar, el tamaño de la muestra es pequeño respecto a las cohortes internacionales. De todas maneras, es un número considerable de pacientes comparado a la experiencia de otros centros similares de Latinoamérica. Otra limitación a considerar es que fue un trabajo unicéntrico desarrollado en un centro monovalente cardiovascular de alta complejidad, con un volumen intermedio de asistencias de ECMO VA por lo que los resultados probablemente no sean extrapolables a otras instituciones de la región ni sean representativos de la realidad nacional.

En conclusión, luego de la implementación del *ECMO Team*, si bien hubo una tendencia favorable en términos de sobrevida intrahospitalaria y destete del ECMO VA de los pacientes asistidos por CC o PCR refractario, no se observó una diferencia estadísticamente significativa. Sin embargo, luego de la creación del equipo multidisciplinario, se observó un aumento significativo del volumen de pacientes asistidos por año. Pos-*ECMO T* se asistieron significativamente mayor número de pacientes por choque cardiogénico, en PCR, y durante más días.

## Fuente de financiamiento:

Autofinanciado
